# Dual-Phase CT Collateral Score: A Predictor of Clinical Outcome in Patients with Acute Ischemic Stroke

**DOI:** 10.1371/journal.pone.0107379

**Published:** 2014-09-11

**Authors:** Na-Young Shin, Kyung-eun Kim, Mina Park, Young Dae Kim, Dong Joon Kim, Sung Jun Ahn, Ji Hoe Heo, Seung-Koo Lee

**Affiliations:** 1 Department of Radiology, Severance Hospital, Yonsei University College of Medicine, Seoul, Korea; 2 Department of Neurology, Severance Hospital, Yonsei University College of Medicine, Seoul, Korea; INSERM U894, Centre de Psychiatrie et Neurosciences, Hopital Sainte-Anne and Université Paris 5, France

## Abstract

**Background and Purpose:**

The presence of good collaterals on CT angiography (CTA) is a well-known predictor for favorable outcome in acute ischemic stroke. Recently, multiphase CT has been introduced as a more accurate method in assessing collaterals. The aim of this study was to assess the ability of dual-phase CT to evaluate collateral status and predict clinical outcome.

**Methods:**

Forty-three patients who underwent both dual-phase CT and transfemoral cerebral angiography (TFCA) for occluded intracranial internal carotid artery (ICA) and/or middle cerebral artery (M1 segment) were recruited from a prospectively collected database. The collateral status on dual-phase CT was graded by using a 4-point scale: grade 0 = no collaterals; 1 = some collaterals with persistence of some defects; 2 = slow but complete collaterals; and 3 = fast and complete collaterals. Univariate and multivariate analysis were performed to define the independent predictors for favorable outcome at 3 months.

**Results:**

Dual-phase CT collateral status (ρ = 0.744) showed higher correlation with TFCA collateral status than CTA collateral status (ρ = 0.596) and substantial interobserver agreement (weighted *κ* = 0.776). In the univariate analysis, age, history of hypertension, collateral scores on CTA, dual-phase CT, and TFCA, occlusion in intracranial ICA, final infarct volume, and symptomatic hemorrhage were significantly associated with outcome. Among them, only the dual-phase CT collateral score was an independent predictor for favorable outcome (OR = 26.342 (2.788–248.864); *P* = 0.004) in the multivariate analysis.

**Conclusions:**

The collateral status on dual-phase CT can be a useful predictor for clinical outcome in acute stroke patients, especially when advanced CT techniques are not available in emergent situations.

## Introduction

The presence of good collateral circulation to the ischemic territory is a notable predictor for favorable long term clinical outcome as well as small infarct size and good response to treatment in patients with proximal intracranial arterial occlusion [1]–[6]. These beneficial roles may be attributable to preserve downstream perfusion distal to occluded vessels, to permit access of thrombolytic materials to the distal end of the clot by retrograde flow, and to augment washout of emboli in distal arteries [7]–[9].

Transfemoral cerebral angiography (TFCA) has been considered as a gold standard to assess collateral status owing to its temporal and spatial resolution. However, global evaluation of pial collateral flow requires catheterization of multiple vessels and, as an invasive investigation with recognized complication rate, is usually only performed when IA treatment is indicated. To aid selection of treatment strategy or to prognosticate, a method of collateral assessment for all stroke patients, not just for those undergoing intra-arterial therapy is warranted. Furthermore, accurate non-invasive assessment of collateral status prior to endovascular therapy would also be advantageous. Therefore, noninvasive assessment of collateral status before IA treatment or in patients who are not candidates for IA treatment may be helpful to select the treatment strategy and predict long term outcome.

Because of short scan times and easy accessibility, computed tomography (CT) is used as the first imaging modality in most patients with acute ischemic stroke. Diverse collateral scoring methods have been suggested in the last decades using CT angiography source images (CTA-SI) [4], [5], [7], [10]–[14] and CTA maximum intensity projection (MIP) images [6], [12], [15], [16]. Although there is no standard scoring system, CTA allows prediction of clinical outcome with moderate to excellent interobserver agreement [11]–[13], [16], [17]. To overcome limitations of CTA such as absence of temporal information from single phase acquisition and possible underestimation of collateral status from faster image acquisition, multi- [18], [19] or tri-phase CT [20] and perfusion CT [3], [21] have been proposed for assessing collateral status. At our institution, the routine CT protocol consists of noncontrast CT (NCCT), CTA, and delayed contrast enhanced CT (CECT). We empirically found that dual-phase CT composed of CTA and delayed CECT is helpful for predicting clinical outcome in patients with acute stroke. Therefore, the aim of this study was to assess the ability of dual-phase CT to evaluate collateral status and predict clinical outcome.

## Materials and Methods

### Patients

The patients were retrospectively selected from a prospectively collected neurointerventional database in a single institution. From February 2011 to June 2013, we recruited consecutive patients who underwent TFCA for possible thrombolysis/mechanical thrombectomy for acute ischemic stroke and met the following inclusion criteria: (1) occlusion of the intracranial ICA or M1 segment; (2) time from symptom onset to admission <6 hours; and (3) presence of a standard local CT protocol including NCCT, CTA, and delayed CECT. Exclusion criteria included bilateral anterior circulation occlusion, posterior circulation occlusion, significant stenosis in contralateral anterior circulation, and past history of stroke.

Clinical data were collected from a prospectively recorded stroke registry database. Type of stroke etiology was classified using Trial of Org 10172 in Acute Stroke Treatment (TOAST) criteria [22]. Functional outcome was assessed using the modified Rankin Scale score (mRS) at 3 months and classified as favorable (≤2) and unfavorable (>2). This study was approved by Severance hospital Institutional Review Board. The Clinical Research Ethics Committee waived the need for written informed consent from the participants because the data released from the hospital database were analyzed anonymously.

### Image Acquisition

CT images were obtained on a 64 multi-detector row CT system (Sensation 64; Siemens, Erlangen, Germany). The sequential axial 3-mm NCCT was performed first with the following parameters from the skull base to the vertex: 120 kVp, 300 mAs, field of view (FOV) of 25 cm, feed/rotation 18 mm, 30×0.6 mm collimation and a H30s medium reconstruction kernel. Each of the conventional axial CT images was subsequently reconstructed into 1.2 mm slices and 1.2 mm increments. A nonionic contrast agent was administered followed by a 40 ml saline chaser at a rate of 4 ml/s by using a power injector. A bolus tracking technique was used with a marker placed at the ascending aorta, triggering at 100 Hounsfield Units (HU) above baseline with a 7 s delay prior to image acquisition. CTA from the aortic arch to the vertex was performed with the following parameters: 120 kVp, 160 mA, FOV of 18–20 cm, 0.33 s per rotation, 0.8 pitch, and a 0.6-mm section thickness. Delayed CECT was performed with the same parameters as NCCT 40 s after contrast injection. The effective dose which was calculated by the CTDI volume x the scan range x the conversion factor was 1.8 mSv for NCCT and CECT, respectively, 0.9 mSv for the cranial portion of CTA, and 3.0 mSv for the cervical portion of CTA. Therefore the total effective dose was 5.7mSv.

TFCA was performed using biplane digital subtraction angiograms (Allura Xper FD20/20; Philips Healthcare, Best, Netherlands) in all patients for the purpose of IA thrombolysis/thrombectomy via bilateral ICAs and the dominant vertebral artery.

Routine follow-up MRI was obtained 1 day after the onset of symptoms with the 3-Tesla MR system (Achieva; Philips Healthcare, Best, Netherlands). DWI was performed with single shot echo planar imaging and parameters were as follows: b-value 1000 s/mm2, TR/TE 6500–7000/65–78 ms, slice thickness 3 mm, FOV 230 mm, matrix 128×128, and NEX 1.

### Image Analysis

#### Imaging studies at admission

*CT:* The extent of ischemic lesions on NCCT scan images at admission was rated according to the Alberta Stroke Programme Early CT Score (ASPECTS) [23].

**Figure 1:**
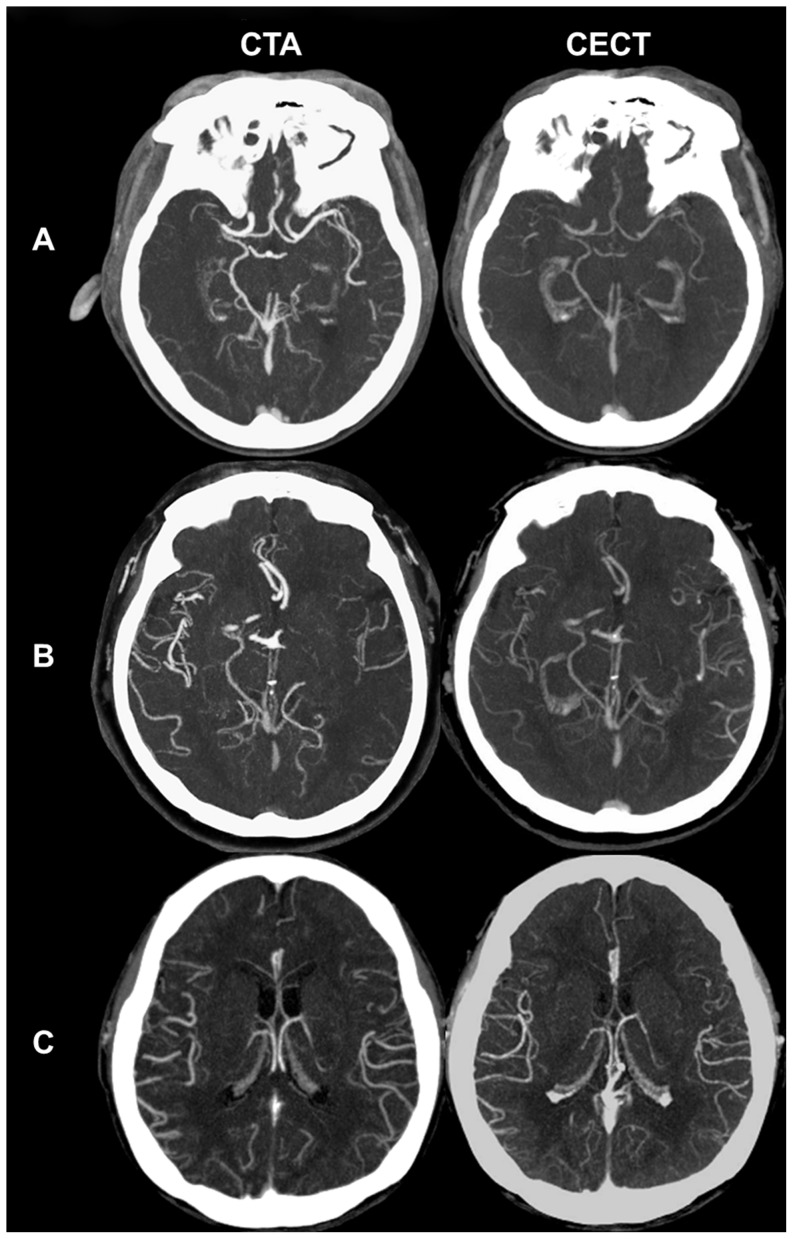
Representative images of collateral status on dual-phase CT. A, Some collaterals to the right MCA territory on CTA with persistence of some defects on CECT (Grade 1). B, Collaterals in part of the left MCA territory on CTA with complete filling on CECT (Grade 2). The collaterals show lower attenuation on CTA with equal to higher attenuation on CECT than unaffected vessels, suggesting slow inflow and washout of collaterals. C, Collaterals in the entire right MCA territory on both CTA and delayed CECT (Grade 3). The collateral vessels show attenuation similar to unaffected vessels on both phase images, suggesting fast velocity of collaterals.

Collaterals on dual-phase CT were graded on CTA and delayed CECT axial MIP images by using a 4-point scale [19]: Grade 0 = no collaterals to the occluded arterial territory; 1 = some collaterals to the occluded arterial territory with persistence of some defects; 2 = slow but complete collaterals (detectable only on delayed CECT) without defects; and 3 = fast and complete collaterals (detectable on both CTA and delayed CECT; Figure 1). Collateral vessel scores were assessed on CTA-SI images using a scale of 0–3: 0 = absence of collateral supply to the occluded vascular territory; 1 = collateral supply filling <50% but >0% of the occluded vascular territory; 2 = collateral supply filling >50% but <100% of the occluded vascular territory; and 3 = collateral supply filling 100% of the occluded vascular territory [6]. The collateral scores on both dual-phase CT and CTA were evaluated independently by two neuroradiologist (N.Y.S. with 4 years of experience and M.P. with 1 year of experience) blinded to the clinical data. Disagreements were settled by consensus.

Occluded segments were recorded based on the location of the non-enhanced segment on delayed CECT as follows [24]: intracranial ICA; proximal M1 segment = occlusion at or proximal to the lenticulostriate arteries; and distal M1 segment = occlusion distal to the lenticulostriate arteries before MCA genu. The thrombus length was measured quantitatively on CECT curved multiplanar reformatted (MPR) images by a neuroimaging fellow (K.E.K. with 1 year of experience) blinded to the clinical data.

*TFCA:* The collateral flow grading system suggested by the Technology Assessment Committees of the American Society of Interventional and Therapeutic Neuroradiology and the Society of Interventional Radiology [24] was used to assess collateral vessels on TFCA: grade 0 = no collaterals to the ischemic region; 1 = slow collaterals to the periphery of the ischemic region with persistence of some defects; 2 = rapid collaterals to the periphery of the ischemic region with persistence of some defects; 3 = slow but complete collaterals to the ischemic bed by the late venous phase; and 4 = complete and rapid collaterals. The assessment of collateral flow was performed through arterial and late venous phases considering all possible collaterals from bilateral carotid and dominant vertebral angiography.

TICI score was recorded to assess recanalization using a 5-point scale [24]: 0 = no perfusion; 1 = penetration of contrast material beyond the occluded segment with minimal perfusion; 2a = partial perfusion (<2/3) of the entire vascular territory; 2b = complete but slow perfusion of the entire vascular territory; and 3 = complete perfusion and rapid clearance comparable to the uninvolved vascular territory. TICI 2b and 3 were considered recanalization.

#### Imaging outcome

The final infarct volume was measured on diffusion weighted images of 1-day follow-up MRI [25] using semiautomatic segmentation. The hemorrhagic transformation on follow-up CT or MRI was classified into hemorrhagic infarction or parenchymal hematoma and determined if it was symptomatic hemorrhage according to ECASS criteria [26].

### Statistics

Nonparametric tests were used for non-normally distributed data. Baseline characteristics were compared according to collateral status on dual-phase CT. Continuous variables are reported as mean ± SD or as the median (interquartile range (IQR)) and were compared using one-way analysis of variance or the Kruskal-Wallis test. Categorical variables are reported as numbers (proportions, %) and were compared using the Fisher's exact test. Post hoc analysis was also performed using the Students' *t*-test, Mann-Whitney *U* test, chi-square test, or Fisher's exact test where appropriate with correction for multiple comparisons.

Spearman's rank correlation was used to assess the correlation between collateral status on dual-phase CT or CTA and collateral status on TFCA. Interobserver agreement for assessment of collateral status on CT was determined using weighted Kappa (*κ*) statistics [27]. The level of agreement was defined as follows: *κ*≤0.2, poor; 0.2<*κ*≤0.4, fair; 0.4<*κ*≤0.6, moderate; 0.6<*κ*≤0.8, substantial, and 0.8<*κ*, good.

To test the association between various clinical and imaging characteristics including collateral status on dual-phase CT and clinical outcome, univariate logistic regression was performed. Multivariate logistic regression with backward elimination (probability value for elimination of 0.1) was used to identify independent factors for favorable outcome. Variables significantly associated with a favorable outcome in the univariate analysis (*P*<0.05) were included in the multivariable model. To assess independent predictors before IA treatment, multivariate analysis with clinical and CT variables was also performed. Receiver operating curve (ROC) comparison was performed to compare the prediction ability of models containing either dual-phase CT collateral (model 1), CTA collateral (model 2), or TFCA collateral (model 3) scores.

All statistical analyses were performed using SAS, version 9.2 (SAS Institute, Cary, NC, USA) and SPSS, version 19 software (IBM Corp., Armonk, NY, USA). ROC curve comparison was performed by using MedCalc, Version 9.3.9.0 software (MedCalc software, Mariakerke, Belgium). *P* values less than 0.05 were considered statistically significant.

## Results

Among 166 patients who underwent TFCA for possible thrombolysis/mechanical thrombectomy for acute ischemic stroke, 123 patients were excluded due to posterior circulation occlusion (n = 34), anterior cerebral arterial occlusion (n = 4), occlusion distal to the M1 segment (n = 41), proximal ICA occlusion only (n = 12), old territorial infarcts (n = 8), multiple territorial infarcts due to cardioembolism (n = 2), significant stenosis in the contralateral M1 segment (n = 1), moyamoya disease (n = 1), and only NCCT acquisition (n = 20). Therefore, a total of 43 patients were recruited for this study. The patients underwent IA treatment with stent-assisted thrombectomy (SAT; n = 29), Penumbra system (n = 1), both SAT and Penumbra system (n = 4), stent placement after mechanical thrombectomy (n = 4), Penumbra system with urokinase infusion (n = 2), and urokinase infusion only (n = 1).

**Figure 2:**
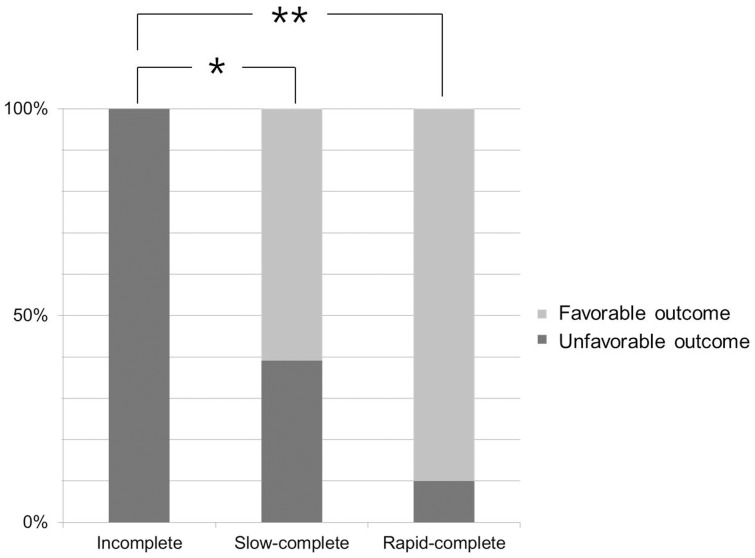
Clinical outcome according to collateral status. All patients with incomplete collaterals showed unfavorable outcome at 3 months. Patients with complete collaterals had favorable clinical outcome more frequently than did patients with incomplete collaterals. * *P* = 0.003; ** *P*<0.001

Patient demographic and clinical data according to collateral status on dual-phase CT are summarized in Table 1. Ten (23.3%) patients were grouped as grade 1 (incomplete collaterals); 23 (53.5%) as grade 2 (slow-complete collaterals); and 10 (23.3%) as grade 3 (rapid-complete collaterals). No patients were classified as grade 0 (no collaterals). There was no statistical difference in the time from symptom onset to CT scan between there groups (*P* = 0.415). Patients with incomplete collaterals were older than those with rapid-complete collaterals (*P* = 0.008). Patients with less collaterals had hypertension more often, although other cardiovascular risk factors did not show differences in the prevalence across different collateral patterns. The pattern of leptomeningeal collaterals on dual-phase CT was also associated with the TOAST classification of stroke: cardioembolic infarcts were observed more often in patients with less collaterals than those with large arterial infarcts. Less collaterals were associated with lower ASPECTS on NCCT (*P* = 0.002) and the presence of intracranial ICA occlusion (*P* = 0.018). Final infarct volume was significantly larger in patients with incomplete collaterals (91.1 (IQR, 49.1–183.2) ml) than that in patients with slow-complete (25.0 (IQR, 10.3–76.7) ml) and rapid-complete (12.3 (IQR, 7.5–27.8) ml) collaterals on dual-phase CT *(P = *0.012 and *P = *0.021, respectively). Clinical outcome was also more favorable in patients with complete collateral patterns (Figure 2). However, there was no significant difference in recanalization rate or hemorrhagic transformation according to collateral pattern.

**Table 1:** Demographic characteristics according to dual-phase collateral status.

	Collateral status on dual-phase CT				
	Incomplete (G1) (n = 10)	Slow-complete (G2) (n = 23)	Rapid-complete (G3) (n = 10)	*P* value	Post-hoc analysis
Age, y, mean ± SD	75.9±6.8	70.1±10.7	61.1±11.4	0.010*	G1>G3 (*P* = 0.008)
Sex, n, M:F	4:3	13:10	6:4	0.974‡	
Risk factors					
Hypertension, n (%)	9 (90.0)	14 (60.9)	1 (10.0)	0.001‡	G1>G3 (*P* = 0.002)
Diabetes, n (%)	4 (40.0)	5 (21.7)	0 (0.0)	0.109‡	
Hypercholesterolemia, n (%)	3 (30.0)	2 (8.7)	0 (0.0)	0.147‡	
Smoking, n (%)	6 (60.0)	8 (34.8)	6 (60.0)	0.302‡	
Coronary artery disease, n (%)	3 (30.0)	10 (43.5)	3 (30.0)	0.763‡	
Clinical measures at presentation					
SBP, mmHg, median (IQR)	134.0 (111.0–161.0)	135.0 (120.0–146.5)	138.5 (112.0–149.0)	0.947†	
DBP, mmHg, mean ± SD	78.5 mmHg	76.3 mmHg	74.9 mmHg	0.680*	
Glucose, mmol/L, median (IQR)	7.25 (6.9–8.6)	6.7 (5.7–8.3)	7.1 (5.9–8.9)	0.490†	
NIHSS score, median (IQR)	16.5 (12.0–19.0)	17.0 (14.3–19.0)	14.0 (13.0–19.0)	0.210†	
TOAST classification				0.015‡	
Large artery, n (%)	1 (10.0)	2 (8.7)	6 (60.0)		
Cardioembolic, n (%)	7 (70.0)	14 (60.9)	4 (40.0)		
Undetermined, n (%)	2 (20.0)	7 (30.4)	0 (0.0)		
CT					
Time from symptom onset to CT, min, mean ± SD	170.9±106.6	209.0±91.1	170.0±87.1	0.415*	
ASPECTS, mean ± SD	6.3±1.8	7.6±1.2	8.5±1.0	0.002*	G1<G2 (*P* = 0.038), G1<G3 (*P* = 0.002)
Occluded segment					
Intracranial ICA, n (%)	5 (50.0)	4 (17.4)	0 (0.0)	0.018‡	
Proximal M1, n (%)	7 (70.0)	16 (69.6)	7 (70.0)	1.000‡	
Distal M1, n (%)	8 (80.0)	20 (87.0)	8 (80.0)	0.753‡	
Thrombus length, mm, median (IQR)	19.3 (11.9–25.5)	15.8 (8.6–18.5)	11.0 (7.3–18.8)	0.298†	
IV tPA					
IV tPA, n (%)	5 (50.0)	11 (47.8)	8 (80.0)	0.275.	
Time from symptom onset to IV tPA, min, mean ± SD	77.8±20.6	108.6±41.4	91.4±38.2	0.301*	
TFCA					
TICI ≥ 2b, n (%)	5 (50.0)	5 (21.7)	2 (20.0)	0.264	
Time from symptom onset to recanalization	385.3±198.2	375.3±106.3	304.0±103.0	0.447	
Outcome					
Final infarct volume, ml, median (IQR)	91.1 (49.1–183.2)	25.0 (10.3–76.7)	12.3 (7.5–27.8)	0.047	G1>G2 (*P* = 0.012), G1>G3 (*P* = 0.021)
Symptomatic hemorrhage	3 (30.0)	3 (13.0)	1 (10.0)	0.537	
parenchymal hematoma	5 (50.0)	4 (17.4)	2 (20.0)	0.199	
3m-mRS ≤ 2	0 (0.0)	14 (60.9)	9 (90.0)	<0.001	G1<G2 (*P* = 0.003), G1<G3 (*P*<0.001)

ASPECTS indicates Alberta Stroke Program Early CT Score; DBP, diastolic blood pressure; IQR, Interquatile range; mRS, modified Rankin Score; NIHSS, National Institutes of Health Stroke Scale; SBP indicates systolic blood pressure; TICI, thrombolysis in cerebral infarction; TOAST, Trial of Org 10172 in Acute Stroke Treatment; and tPA, tissue plasminogen activator.

* one-way ANOVA; † Kruskal Wallis test; ‡ Fisher's exact test

The dual-phase collateral scores showed higher correlation with collateral status on TFCA (ρ = 0.744; *P*<0.001) than that of the CTA collateral scores (ρ = 0.596; *P*<0.001). Interobserver agreement for the collateral scoring on dual-phase CT [weighted *κ* = 0.776 (0.619–0.933)] was substantial and better than that on CTA [weighted *κ* = 0.475 (0.285–0.665)].

Univariate analysis of patients with good and poor clinical outcome is presented in Table 2. Younger age (*P* = 0.040), absence of history of hypertension (*P* = 0.022), higher collateral scores on CTA (*P* = 0.002), dual-phase CT (*P* = 0.004), and TFCA (*P* = 0.002), absence of occlusion in intracranial ICA (*P* = 0.048), smaller final infarct volume (*P* = 0.003), and absence of symptomatic hemorrhage (*P* = 0.048) were all significantly associated with favorable clinical outcome. Multivariate analysis of all significant variables using backward elimination showed that only the collateral score on dual-phase CT [odds ratio (OR) = 26.342 (2.788–248.864); *P* = 0.004] was an independent predictor for favorable outcome with adjustment for NIHSS scores at admission [OR = 0.834 (0.679–1.025); *P* = 0.085]. Moreover, multivariate analysis with significant clinical and imaging variables before IA treatment also showed the dual-phase CT collateral score [OR = 27.117 (1.519–484.258); *P* = 0.025] as an independent predictor for clinical outcome even after adjustment of age, presence of hypertension, NIHSS score at admission, ASPECTS, and occlusion of intracranial ICA. Meanwhile, the CTA collateral score in model 2 was not an independent predictor (*P* = 0.062) unlike dual-phase CT collateral score in model 1. Although the TFCA collateral score was also an independent predictor [OR = 2.996 (1.138–7.890); *P* = 0.026], the area under the curve (AUC) of model 3 (0.874) was lower than that of model 1 (0.922). However, it failed to achieve a level of statistical significance (*P* = 0.206; Table 3).

**Table 2:** Univariate analysis for favorable clinical outcome (mRS ≤2) at 3 months.

	Favorable (n = 23)	Unfavorable (n = 20)	Univariate analysis		
			Odds ratio	95% CI	*P* value
Age, y, mean ± SD	66.1±10.2	73.4±11.0	0.934	0.874–0.997	0.040
Male, n (%)	16 (69.6)	9 (45.0)	2.794	0.800–9.760	0.107
Risk factors					
Hypertension, n (%)	9 (39.1)	15 (75.0)	0.214	0.058–0.797	0.022
Diabetes, n (%)	4 (17.4)	5 (25.0)	0.632	0.144–2.771	0.542
Hypercholesterolemia, n (%)	1 (4.3)	4 (20.0)	0.182	0.019–1.785	0.143
Smoking, n (%)	12 (52.2)	8 (40.0)	1.636	0.487–5.500	0.426
Coronary artery disease, n (%)	11 (47.8)	5 (25.0)	2.749	0.748–10.102	0.128
Clinical measures at admission					
SBP, mmHg, median (IQR)	135.0 (114.0–144.0)	140.5 (113.5–163.5)	0.980	0.956–1.005	0.114
DBP, mmHg, mean ± SD	73.5±11.0	79.9±17.3	0.967	0.924–1.013	0.157
Glucose, mmol/L, median (IQR)	6.7 (103.0–154.3)	7.25 (121.0–152.5)	1.028	0.793–1.334	0.833
NIHSS score, median (IQR)	15.0 (13.0–16.8)	18.0 (15.5–19.0)	0.873	0.747–1.020	0.088
Delays					
Time from symptom onset to IV tPA, min, mean ± SD	96.8±39.5	96.4±36.7	1.000	0.978–1.023	0.983
Time from symptom onset to CT, min, mean ± SD	198.0±95.7	183.0±93.2	1.002	0.995–1.008	0.596
Time from symptom onset to IA treatment, min, mean ± SD	268.2±107.9	259.2±113.8	1.001	0.995–1.006	0.785
TOAST classification					
Large artery, n (%)	7 (30.4)	2 (10.0)	Reference		0.286
Cardioembolic, n (%)	12 (52.2)	13 (65.0)	0.264	0.046–1.528	0.137
Undetermined, n (%)	4 (17.4)	5 (25.0)	0.229	0.029–1.774	0.158
CT					
ASPECTS, mean ± SD	8.0±0.9	6.9±1.8	1.908	1.135–3.209	0.015
CTA Collateral score	2.0 (2.0–3.0)	1.0 (1.0–2.0)	6.077	1.926–19.167	0.002
0, n (%)	0 (0.0)	0 (0.0)			
1, n (%)	4 (17.4)	14 (70.0)			
2, n (%)	12 (52.2)	5 (25.0)			
3, n (%)	7 (30.4)	1 (5.0)			
Dual-phase CT collateral score	2.4±0.5	1.6±0.6	19.436	2.581–146.371	0.004
0, n (%)	0 (0.0)	0 (0.0)			
1, n (%)	0 (0.0)	10 (50.0)			
2, n (%)	14 (60.9)	9 (45.0)			
3, n (%)	9 (39.1)	1 (5.0)			
Occluded segment					
Intracranial ICA, n (%)	2 (8.7)	7 (35.0)	0.177	0.032–0.985	0.048
Proximal M1, n (%)	14 (60.9)	16 (80.0)	0.389	0.098–1.544	0.179
Distal M1, n (%)	20 (87.0)	16 (80.0)	1.667	0.325–8.549	0.540
Thrombus length, mm, median (IQR)	11.4 (7.4–17.6)	17.9 (10.4–22.8)	0.938	0.871–1.011	0.093
Treatment					
IV tPA only, n (%)	3 (13.0)	0 (0.0)	Reference		0.639
IA only, n (%)	8 (34.8)	11 (55.0)	0.000	0.000	0.999
IV tPA and IA, n (%)	12 (52.2)	9 (45.0)	0.000	0.000	0.999
TFCA					
TFCA collateral score	3.0 (2.3–4.0)	2.0 (1.0–2.0)	3.092	1.537–6.221	0.002
0, n (%)	0 (0.0)	0 (0.0)			
1, n (%)	2 (8.7)	8 (40.0)			
2, n (%)	4 (17.4)	8 (40.0)			
3, n (%)	6 (26.1)	2 (10.0)			
4, n (%)	11 (47.8)	2 (10.0)			
TICI ≥ 2b, n (%)	4 (17.4)	8 (40.0)	0.316	0.078–1.282	0.107
Time from symptom onset to recanalization, min, mean ± SD	352.0±110.6	383.0±152.3	0.998	0.993–1.003	0.998
Imaging outcome					
Final infarct volume, ml, median (IQR)	13.7 (7.2–25.0)	80.7 (34.0–144.8)	0.965	0.943–0.988	0.003
Symptomatic hemorrhage, n (%)	1 (4.3)	6 (30.0)	0.106	0.012–0.977	0.048
Parenchymal hematoma, n (%)	3 (13.0)	8 (40.0)	0.225	0.050–1.016	0.052

ASPECTS indicates Alberta Stroke Program Early CT Score; DBP, diastolic blood pressure; IQR, Interquatile range; mRS, modified Rankin Score; NIHSS, National Institutes of Health Stroke Scale; SBP indicates systolic blood pressure; TFCA, transfemoral cerebral angiography; TICI, thrombolysis in cerebral infarction; TOAST, Trial of Org 10172 in Acute Stroke Treatment; and tPA, tissue plasminogen activator.

**Table 3:** Multivariate analysis for favorable clinical outcome (mRS ≤2) at 3 months.

	Model 1			Model 2			Model 3		
	Odds ratio	95% CI	*P* value	Odds ratio	95% CI	*P* value	Odds ratio	95% CI	*P* value
Age	0.969	0.880–1.067	0.520	0.953	0.877–1.035	0.250	0.958	0.879–1.044	0.332
Hypertension	2.682	0.294–24.508	0.382	1.435	0.187–10.993	0.728	1.164	0.171–7.939	0.877
NIHSS score at admission	0.814	0.648–1.023	0.077	0.868	0.731–1.029	0.103	0.826	0.678–1.006	0.058
ASPECTS	1.504	0.675–3.351	0.318	1.515	0.793–2.893	0.208	1.480	0.734–2.984	0.273
Occlusion of intracranial ICA	0.518	0.057–4.679	0.558	0.273	0.032–2.318	0.234	0.245	0.025–2.371	0.225
Dual-phase CT collateral score	27.117	1.519–484.258	0.025	-	-	-	-	-	-
CTA collateral score	-	-	-	4.456	0.929–21.365	0.062	-	-	-
TFCA collateral score	-	-	-	-	-	-	2.996	1.138–7.890	0.026
AUC	0.922 (0.798–0.981)			0.880 (0.745–0.959)			0.874 (0.737–0.955)		

ASPECTS indicates Alberta Stroke Program Early CT Score; AUC, area under the curve; ICA, internal carotid artery; and NIHSS, National Institutes of Health Stroke Scale.

## Discussion

The present study suggests collateral status on dual-phase CT may be an independent predictor [OR = 26.342 (2.788–248.864); *P* = 0.004] for clinical outcome in acute ischemic stroke patients who are subjected to IA treatment. Moreover, after adjustment of clinical and CT variables measured before IA treatment, collateral status on dual-phase CT was also an independent predictor for clinical outcome [OR = 25.763 (1.342–494.601); *P* = 0.031]. On the other hand, the CTA collateral score could not independently predict clinical outcome in model 2. Also, although there was no statistical significance, the prediction model with the dual-phase CT collateral score was better than that with TFCA collateral scores.

Leptomeningeal collaterals have been considered as having a beneficial role in patients with acute stroke despite various scoring scales and different imaging modalities [17]. CT, which is noninvasive and widely available in emergency situations, has been used to evaluate collateral status. Above all, CTA-based methods are the most commonly suggested so far [4]–[6], [10]–[14]. However, the prediction power of these methods can be influenced by small differences in time delay from contrast injection to image acquisition as there is no dynamic information on single phase CTA. Before the era of fast CTA acquisition, CTA images were acquired under an almost steady-state condition with maximal enhancement in arteries and tissues. Therefore, the nonenhanced area on this protocol is associated with decreased cerebral blood volume and can be a predictor for cerebral infarction [11]. After the introduction of multidetector CT that has made fast acquisition of CTA possible, slowly perfused areas via collaterals might not be enhanced on CTA images causing overestimation of infarct core [28] and thrombus length [29] as well as underestimation of collateral status [21], [30]. To overcome limitations of single phase CTA, multi- [18], [19] and tri-phase [20], [31] CT and perfusion CT [3], [21] have been suggested as a tool to assess collateral status; but the additional value of these advanced techniques over single phase CTA to predict clinical outcome lacks confirmation. Moreover, lack of ubiquitousness of these advanced sequences and post processing techniques, insufficient scan coverage, and potential radiation hazard caused by additional CT scans might be problems in daily clinical practice.

A previous study using tri-phase CT [31] graded collateral scores on each phase image and focused on identifying the best phase to predict infarct volume. A recent study with time-resolved assessment of collateral status on perfusion CT [21] also scored collateral status on early and peak phases and temporal MIP reconstruction images which were obtained by fusing contrast enhancement across the whole duration of the 4-dimensional CTA. This study classified patients into incomplete, slow-complete, and rapid-complete collateral flow groups by integrating dynamic information from various phase images. In addition, they also showed that complete collateral flow groups achieve favorable clinical outcome more often; however, the effective dose of this CT protocol was 8.5 mSv excluding NCCT. Kim et al. [19] obtained multiphase CT and evaluated collateral status on mid (23 and 32 s after contrast injection) and late (41 and 50 s after contrast injection) phase CECT images by using the same scales that were used in our study. They found high correlation between collateral status on these two phase images and TFCA. However, analysis of the effect of collateral status on multiphase CT on clinical outcome was not conducted. It is worth noting that the time delay between contrast injection and image acquisition of mid and late phases were similar to those of CTA and delayed CECT in the present study. Thus, supporting that only dual-phase CT with optimized time delay from contrast injection may have sufficient dynamic information to show collateral status.

In this study, collateral scores on dual-phase CT showed better correlation with collateral status on TFCA and higher interobserver agreement than did collateral scores on single phase CTA. Also, as mentioned above, collateral status on dual-phase CT was an independent predictor for clinical outcome; however, collateral scores on single phase CTA and even TFCA were not. On dual-phase CT, detection of collateral defects on CTA and delayed CECT was needed. On the other hand, additional detection on the degree (smaller or larger than 50% of the occluded territory) of collateral extent was required on single phase CTA. Therefore, the observers might grade collateral status more consistently on dual-phase CT. Both dual-phase CTA and TFCA provide dynamic information of collaterals; however, it is difficult to evaluate pial collaterals from whole cerebral circulation by TFCA, so the prediction power for clinical outcome might be lower than that of dual-phase CT.

Among the patients with complete collaterals on dual-phase CT, patients with rapid-complete collaterals had higher ASPECT scores at admission (7.6 vs. 8.5), smaller final infarct volume (25.0 ml vs. 12.3 ml), and more frequent favorable clinical outcome (60.9% vs. 90%) than patients with slow-complete collaterals. Although there was no significant differences between the two groups as reported in a previous study [21], the number of patients may have been too small to reach a statistically significant conclusion. Therefore, prospective studies with larger sample size and an optimized CT protocol are warranted to establish the influence of collateral velocity on clinical outcome.

There were several limitations in our study. First, this study was a retrospective study although the patients were recruited from a prospectively collected database. Also a small number of patients included in the analysis might cause a wide range of 95% CI of OR of dual-phase CT collateral status and its overestimation. Therefore, caution is needed in generalizing the results of this study. Second, more than 50% of patients were treated with IV thrombolysis. As a result of the IV treatment, potential confounding factors may have influenced the assessment of collateral status on TFCA and prediction of clinical outcome. There might be difference in thrombus extent and the collateral status between CT and TFCA. Third, there is the possibility that selection bias may exist. Patients who achieved recanalization only after IV treatment or had substantial ischemic change larger than 1/3 of the middle cerebral artery (MCA) territory were not subjected to IA treatment and excluded from this study. We also excluded considerable number of patients according to the exclusion criteria. However, our results could be applied to a relatively homogenous patient group with occlusion of distal ICA or M1 segments who are indicated for IA treatment. Fourth, we did not perform multiphase CT or perfusion CT, so direct comparison between these advanced techniques and dual-phase CT in predicting clinical outcome was not performed. Finally, our routine CT protocol was not optimized to assess collateral status in patients with acute ischemic stroke. According to a previous study using time-resolved CTA [21], alteration of time delay from contrast injection to image acquisition has the potential to impair outcome prediction. Therefore, efforts to establish an appropriate CT protocol and future studies with this optimized CT protocol in a larger scale are required.

## Conclusions

In conclusion, dual-phase CT is feasible for assessing collateral status in patients with acute ischemic stroke, showing substantial interobserver agreement. Collateral status on dual-phase CT can also be a useful predictor for clinical outcome in acute stroke patients subjected to IA treatment, especially when advanced CT techniques are not available in emergency situations.
